# Role of Somatostatin Signalling in Neuroendocrine Tumours

**DOI:** 10.3390/ijms23031447

**Published:** 2022-01-27

**Authors:** Olesja Rogoza, Kaspars Megnis, Marija Kudrjavceva, Aija Gerina-Berzina, Vita Rovite

**Affiliations:** 1 Latvian Biomedical Research and Study Centre, Ratsupites Str 1-k1, LV-1067 Riga, Latvia; olesja.rogoza@biomed.lu.lv (O.R.); kaspars.megnis@biomed.lu.lv (K.M.); 2 Pauls Stradins Clinical University Hospital, Pilsonu Str 13, LV-1002 Riga, Latvia; m.kudrjavceva93@gmail.com (M.K.); a.gerina@inbox.lv (A.G.-B.)

**Keywords:** neuroendocrine tumour, somatostatin, somatostatin receptor, somatostatin analogue, octreotide, lanreotide, PRRT

## Abstract

Somatostatin (SST) is a small peptide that exerts inhibitory effects on a wide range of neuroendocrine cells. Due to the fact that somatostatin regulates cell growth and hormone secretion, somatostatin receptors (SSTRs) have become valuable targets for the treatment of different types of neuroendocrine tumours (NETs). NETs are a heterogeneous group of tumours that can develop in various parts of the body, including the digestive system, lungs, and pituitary. NETs are usually slow growing, but they are often diagnosed in advanced stages and can display aggressive behaviour. The mortality rate of NETs is not outstandingly increased compared to other malignant tumours, even in the metastatic setting. One of the intrinsic properties of NETs is the expression of SSTRs that serve as drug targets for SST analogues (SSAs), which can delay tumour progression and downregulate hormone overproduction. Additionally, in many NETs, it has been demonstrated that the SSTR expression level provides a prognostic value in predicting a therapeutic response. Furthermore, higher a SSTR expression correlates with a better survival rate in NET patients. In recent studies, other epigenetic regulators affecting SST signalling or SSA–mTOR inhibitor combination therapy in NETs have been considered as novel strategies for tumour control. In conclusion, SST signalling is a relevant regulator of NET functionality. Alongside classical SSA treatment regimens, future advanced therapies and treatment modalities are expected to improve the disease outcomes and overall health of NET patients.

## 1. Introduction

Neuroendocrine tumours (NETs) represent a heterogeneous group of neoplasms and can originate from neuroendocrine cells in any organ of the body [1]. For all NETs, overall survival rates are approximately 55% and 45% five and ten years after diagnosis, respectively [2]. The classification and clinical picture of each NET differs based on the organ of origin; however, all NETs share the expression of somatostatin receptors (SSTRs), which have become valuable targets for somatostatin analogue (SSA) therapy. Symptom management is the most prevailing therapy in patients with functioning NETs. Here, we compare this approach to other treatment options, while discussing the role and relevance of each.

The purpose of this review is to summarize information about the role of somatostatin signalling in NETs, as well as the development, prognosis, and treatment for these tumours. We also aim to demonstrate novelties in this field and highlight the importance of the somatostatin signalling system as one of most relevant targets for NET therapies.

For the literature analysis, we used the following methodological approach. Peer-reviewed study articles were derived from the National Centre for Biotechnology Information database (https://pubmed.ncbi.nlm.nih.gov/ accessed on 25 November 2021) during the period from September 2021 to November 2021. Each author was responsible for a specific section of an article. In addition to study articles, authors used appropriate book chapters from the available literature [3].

In articles used for the review, the classification of NETs differed due to diverse classification systems (some articles dated back to the early 2000s). In this article, we maintain the classification used in each source rather than reclassifying to avoid any bias of regrouping the tumours. We found the most literature in the area of gastroenteropancreatic tumours, which is also predominant in our review; however, we also include other NET types, such as lung and pituitary tumours, since pituitary tumours share many NET-related properties and serve as targets for SSA. The similarity between NETs is also stressed by the *World Health Organization’s Classification of Tumours of Endocrine Organs*, which was published in 2017 [4].

## 2. Somatostatin Signalling

Somatostatin (SST), also known as the somatotropin release-inhibiting factor (SRIF) or growth hormone-inhibiting hormone (GHIH), is a cyclic peptide that exerts inhibitory effects on the endocrine and exocrine hormone secretion [5,6]. The growth hormone (GH), prolactin (PRL), thyrotropin (TSH), cholecystokinin, gastric inhibitory peptide, neurotensin, motilin, gastrin, secretin, glucagon, insulin, pancreatic polypeptide, and cytokines in immune cells are all inhibited by SST [5,7]. The effects of SST on exocrine hormones include the suppression of amylase secretion from salivary glands; the inhibition of hydrochloric acid, pepsinogen, and intrinsic factors in the gastrointestinal mucosa; the reduced secretion of pancreatic enzymes and bicarbonate, and the reduced secretion of bile from the liver. SST also prevents the absorption of glucose, fat, and amino acids, helps to regulate gastrointestinal motility by delaying late gastric emptying, weakens gallbladder contractions, and lengthens small intestinal transit time. SST also reduces the time between migrating motor complexes and accelerates early stomach emptying. Immunoglobulin production and lymphocyte proliferation have both been found to be inhibited by SST in lymphoid tissues [7].

**Figure 1:**
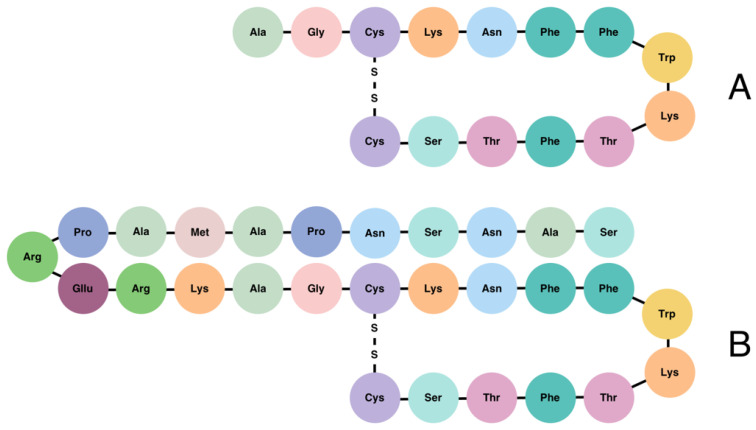
Structure of somatostatin-14 (**A**) and somatostatin-28 (**B**).

SST is produced in many locations of the body, primarily in the pancreas, gastrointestinal (GI) tract, central nervous system (CNS), and hypothalamus [8]. Both isoforms of SST (SST-14 and SST-28) are derived from a 116-amino acid precursor protein, known as pre-prosomatostatin, which is cleaved into 92-amino acid prosomatostatin. To generate SST-14 and SST-28, prosomatostatin undergoes C-terminal post-translational processing, which results in the production of a predominant 14-amino acid molecule as well as a larger, N-terminally extended 28-amino acid form (Figure 1) [9,10]. While the shortest isoform is secreted mainly from the β-cells of the pancreas, the SST-28 is the product of GI cells [11,12]. SST-14 has wide-ranging effects, including the inhibition of GH, TSH, and corticotropin (ACTH) within the pituitary, as well as the inhibition of glucagon and insulin in the pancreas [13]. SST-28 refers to the endogenous pro-form of SST, which regulates the inhibition of the hormones previously mentioned in the context of SST-14 [14]. SST-28 is known to be more potent than SST-14 with respect to its effect on GH, PRL, insulin, glucagon, TSH, and gonadotropins (LH and FSH) secretion [15]. It is estimated that 65% of the circulating SST is produced and secreted by the D-cells of the GI tract, 30% is produced by the CNS (hypothalamus and amygdala), and the remaining 5% is produced by pancreatic β-cells [9,16]. Both SST active forms are stored in secretory granules and have a short (~1 min) bioactive half-life time (t½) once released into the circulation [5,12].

The activity of SST is mediated by its binding to five subtypes of SSTRs, each encoded by five different genes segregated on chromosomes 14, 17, 22, 20, and 16, respectively [17]. The SST receptor subtypes (SSTR1 through SSTR5) share signalling pathways and structural features [18]. Two isoforms of SSTR2 exist (SSTR2A and SSTR2B), and are produced via alternative splicing [19,20]. These variants of SSTR2 differ in the length of their C-terminal cytoplasmic tails as well as their ability to couple to adenylyl cyclase; as a result, SSTR2A and SSTR2B may activate alternative signal transduction pathways [21,22]. SSTRs belong to the superfamily of G-protein-coupled receptors (GPCRs) and play a crucial role in vertebrate development, metabolism, and growth [23]. GPCRs represent the largest family of human membrane proteins, characterized by a core of seven transmembrane helices that are connected by three extracellular loops (ECLs) and an amino terminus [17,24]. The activation of SSTRs usually results in the inhibition of adenylyl cyclase and the reduction in intracellular Ca^2+^, which result in the inhibition of cell proliferation and the secretion of signalling molecules [25,26]. Firstly, SST activates the SSTR which interacts with the G protein, consisting of α-, β-, and γ-subunits, thereby modulating several downstream second messenger systems. The α-subunit reduces the affinity for guanosine diphosphate (GDP), and because the concentration of guanosine triphosphate (GTP) is higher in the cytoplasm, GDP is, thus, replaced by GTP. Thereafter, the Gα protein dissociates from the receptor and subunit complex and modulates the activity of several intracellular pathways [17].

SSTRs are expressed throughout many tissues of the body, including the hypothalamus and pituitary, GI tract peripheral organs, and pancreas, as well as kidneys, thyroid, lungs, and immune cells. Moreover, SSTR expression has been reported in numerous types of tumours [17]. In the GI tract, SST regulates the release of gastric acid by a negative feedback mechanism of paracrine effects. The feedback pathway involves stomach D-cell SST release in response to direct stimulation by gastrin, and this indirectly inhibits further gastric release from G-cells [27]. Within the hypothalamus, SST indicates the inhibition of GH, LH, and TSH release from the pituitary [28]. SST binds to the β-cells of the pancreas to inhibit voltage-gated calcium channels, resulting in the suppression of the early insulin response to glucose and, thus, downmodulating the storage of energy in adipose tissue [29]. SST suppresses several immune functions, such as lymphocyte proliferation, immunoglobulin production, and the release of proinflammatory cytokines such as interferon-γ (IFNγ) and interleukin-8 (IL-8) [30,31]. The effect of SST under physiological conditions is partially determined by the types of SSTRs expressed on the tissue’s surface [8]. For instance, SSTR2 and SSTR5 have been reported as the most abundantly expressed receptors. Both show inhibitory effects on GH and ACTH within the pituitary gland, on insulin, within the β-cells of the pancreas, and on glucagon-like peptide 1 (GLP-1), IFN-γ, and reduce the secretion of gastric acid [18,26,32]. SSTR2 is extensively expressed in pulmonary endocrine tumours, including typical and atypical carcinoids and non-endocrine lung cancers such as adenocarcinoma and small cell lung cancer [33]. Receptor expression profiles differ between patients and even between tumours within the same patient. SSTR2 is expressed in about 80% of GI tract and pancreatic endocrine tumours according to Reubi and colleagues [3]. Among the tumours, SSTR2A is the most commonly expressed receptor subtype. The expression of SSTR2A has been reported in gastrinomas, insulinomas, gliomas, medulloblastomas, paragangliomas, and neuroblastomas [34,35]. Neuroblastomas are the most common malignancy among children. These types of tumours are typically associated with a high expression of SSTR1 and SSTR2, which usually indicates a good prognosis for patients [36]. SSTR2A correlates with the overall survival rate in patients with medullary thyroid carcinoma and is considered as a favourable prognostic marker in stage IV patients [37]. The activation of SSTR1 shows antisecretory effects on the GH, PRL, and calcitonin, whereas SSTR3 regulates antiproliferative signalling and induces apoptosis in several cell types [32,38]. The role of SSTR4 remains mostly unknown, but it may be linked to the inflammation of the intestine. SSTR4 has been identified as a key player in the inflammatory effects exerted by SST, either through the direct targeting of inflammatory cells or via the indirect modification of cells that synthesize and release pro-inflammatory mediators [39]. Pro-inflammatory mediators are released from capsaicin-sensitive sensory nerve endings during inflammation. These mediators primarily include tachykinins (substance P and neurokinin A) and the calcitonin gene-related peptide, which may be involved in the sympathetic reflex inhibition of GI propulsion, ultimately initiating an inflammatory cascade [39,40]. The expression of SSTR4 has also been detected in the lungs, heart, and placenta [18].

Expression levels of SSTRs have been reported in the majority of NETs and non-neuroendocrine tumour types, including pancreatic NETs (PanNETs), pituitary NETs (PitNETs), and gastroenteropancreatic NETs (GEP-NETs), as well as hepatocellular carcinoma and breast cancer [25,41,42,43]. Since SSTRs are located on the surface of tumour cells, they have the potential to serve as diagnostic markers and be used for SSA treatment strategies [39].

## 3. Neuroendocrine Tumours

NETs are a heterogeneous group of generally slow-growing neoplasms of epithelial origin with variable clinical prognoses and behaviour [44,45]. NETs, not to be confused with neuroendocrine carcinomas (NECs), are believed to originate from hormonally programmed neuroendocrine precursor cells that undergo tumourigenic mutational events. For this reason, NETs mostly consist of well-differentiated neuroendocrine cells. Normally, neuroendocrine cells can be either diffusely distributed in the mucosal membrane, as in the case in the digestive system, or they can form organised cell clusters or organs of endocrine function, such as pancreatic islets or the pituitary gland [1]. Neuroendocrine cells are widely distributed in the human body and, for this reason, NETs can occur in virtually any location. However, NETs occur most commonly in the GI tract, pancreas, and lungs [46].

NETs are generally subdivided by their proliferative activity using the mitotic and/or Ki67 index. G1 NETs are classified by <2 mitoses/10 high-power fields and a Ki-67 index of <3%, G2 NETs are classified by 2–20 mitoses/10 high-power fields or a Ki-67 index of 3–20%. Well-differentiated G3 NETs are classified by >20 mitoses/10 high-power fields or a Ki-67 index of >20%, and poorly differentiated G3 NECs are classified by >20 mitoses/10 high-power fields or Ki-67 and expression alterations of p53 and Rb1 [1,47,48,49]. Further classification depends on tumour location and functionality, and it is not uniform across different centres.

Due to their hormonal origin, NETs can synthesise and secrete cell-type-specific peptide hormones and neuroamines, and, therefore, are characterised as functioning NETs [50]. For example, PitNETs can additionally secrete GH, PRL, or other pituitary hormones, thereby elevating hormone concentrations in the circulation, leading to hormonal dysregulations and characteristic clinical syndromes [51,52]. About 60–90% of PanNETs are non-functioning and do not show significant symptoms. Functioning PanNETs are uncommon and are usually associated with the increased secretion of various hormones, including insulin, gastrin, ghrelin, vasoactive intestinal peptide (VIP), glucagon, and SST [53]. Most GEP-NETs are non-functioning and present moderately late; in turn, functioning tumours cause distinct clinical syndromes resulting from the production of various bioactive peptides or amines. For instance, active gastric NETs (GNETs) are known to secrete histamine, yet the duodenum produces secretin, gastrin, gastric inhibitory polypeptide, and motilin [54]. Furthermore, NETs have the capacity to modify secreted hormones and peptides at the genetic level. For example, gastrin may appear in five different forms in the circulation, due to different splice variants in NETs [55]. Non-functioning NETs are not associated with specific hormonal changes or clinical syndromes. As a result, non-functioning NETs are usually diagnosed in the later stages after the occurrence of symptoms related to tumour mass effects or metastases [51,52]. The liver is the most common site of NET metastasis. Due to improved diagnostic tools and an increase in early diagnosis, the majority of cases at the time of diagnosis are graded as G1; most of these are non-metastatic, but only by a small margin [56].

Biochemical and tissue markers in GEP-NETs are applied for diagnostic, prognostic, and predictive intentions [53,57]. Chromogranin A (CgA) is considered to be one of the most implemented biomarkers in the diagnosis and prognosis of NETs. Despite an overall diagnostic sensitivity of 73% and a specificity of 95%, the use of CgA as a biomarker for NETs has gradually declined in recent years. The accuracy of CgA can vary largely based on the type of NET and it can be falsely elevated in the presence of various conditions, such as atrophic gastritis and liver disease, or in cases involving treatment with proton pump inhibitors [58,59,60,61,62]. Other general markers, including the Neuron-Specific Enolase (NSE) and pancreatic polypeptide (PP), are mainly elevated in poorly differentiated NETs and non-functioning NETs, respectively [57]. The serotonin metabolite 5-hydroxy indole acetic acid (5HIAA) can be measured in urine or blood plasma and is used as a diagnostic and follow-up marker for patients with midgut NETs. However, the specificity of 5HIAA is influenced by the fact that its hypersecretion has also been observed in patients with carcinoid syndrome [63,64,65]. Circulating biomarkers, such as gastrin, insulin, glucagon, SST, and VIP, are specific PanNET biochemical markers used for diagnosis and treatment monitoring [66]. Several novel biomarkers have been discovered to improve the early diagnosis and monitoring of NETs, including programmed death ligand-1 and glucose transporters type 1 within lung NETs (Lu-NETs) and PanNETs, as well as survivin, an inhibitor of apoptosis, in Lu-NETS, PanNETs, and GI-NETs [67]. Circulating tumour cells (CTCs) are a relatively novel biomarker for NETs; the elevation of CTCs is measured based on the expression of the epithelial cell adhesion marker [68,69]. The absence of CTCs strongly correlates with 5HIAA and liver metastases extension, indicating disease progression. Therefore, the absence of CTCs may be considered as a prognostic biomarker [69]. Despite promising results of existing research, additional data and evidence in this regard remain sparse. Further studies are necessary to convincingly demonstrate the clinical usefulness of CTCs as a biomarker for NETs.

Although NETs are mostly sporadic, they can be associated with multiple inherited syndromes, including multiple endocrine neoplasia (MEN) types 1, 2, and 4, as well as Von Hippel–Lindau syndrome, neurofibromatosis 1 (NF1), and tuberous sclerosis. These syndromes are caused by dysregulating mutations in oncogenes, proto-oncogenes, tumour suppressors, or cell cycle regulator genes. As a result, these dysregulations can cause cell tumourous growth, among other symptoms [55,70].

NETs are extensively vascularised tumours. They typically have an increased expression of vascular endothelial growth factor (VEGF) and VEGF receptor (VEGFR) subtypes [47]. Interestingly, VEGF expression has been observed to be higher in well-differentiated NETs, compared to poorer differentiated counterparts. The intratumoural vessel density in NETs is approximately 10-fold higher as compared with carcinomas, which elevates not only NET growth, but also the release of secretory products into the bloodstream [71].

## 4. Somatostatin Signalling in NET Development and Prognostics

Somatostatin signalling is involved in many cell regulatory circuits that can inhibit tumourigenesis. Therefore, shifts in SST signal transduction pathways can significantly contribute to NET development within an affected tissue. The binding of SST to SSTR inhibits the activity of adenylyl cyclase, downregulating the concentration of the second messenger cAMP and following intracellular calcium, leading to a decreased hormone secretion [72,73]. This is particularly important in hormone-producing NETs, where SSA treatment can help to normalise adverse effects of excessive hormone levels in the body. SST signals are also important cell cycle regulators; a ligand binding to SSTR activates protein tyrosine phosphatases SHP-1 and SHP-2, leading to a decreased cell proliferation via the upregulation of cell cycle inhibitors p27 and p21, as well as the inhibition of PI3K/AKT and MAPK, thereby attenuating cell division [18,72]. This property could indicate the possibility to regulate other tumour growth through targeting the SST signalling pathway, especially since SSTRs are expressed also in breast, thyroid, prostate cancer tissues, glioma, hepatocellular carcinoma, and other tumours [74]. Other hallmarks of tumourigenesis, such as angiogenesis and cell migration, are also regulated by SST signalling. The VEGF platelet-derived growth factor (PDGF), insulin-like growth factor, and basic fibroblast growth factor have been shown to enhance the neovascularization and cell growth of tumours [75,76,77,78]. On a cell signalling and functional level, SST and SSTR significantly inhibit hormonal secretion, cell cycle progression, angiogenesis, and cell migration. However, the role of SST signalling and SSTR in NET development, the response to SSA, and prognosis highly depends upon SSTR distribution in different tumour types and additional intrinsic factors of specific tumours [41].

Many studies have assessed the expression of SSTRs in different tumours. The techniques used in these studies include PCR-based methods, immunohistochemistry (IHC), and somatostatin receptor scintigraphy (SRS), each of which has specific advantages and drawbacks (Table 1) [79,80,81,82,83,84,85,86,87]. PCR-based expression evaluation methods remain cost effective, easy to perform, and are scalable. This method is also easily interpreted without highly sophisticated professional experience or background, compared to IHC and imaging. PCR gives bulk representative values for tumour tissue expression levels. However, in several studies, good concordance has been demonstrated between RT-PCR and IHC data [81,88]. On the other hand, IHC and imaging allow for a more precise evaluation of expression levels. However, these techniques are more costly and are highly dependent on available equipment and professional expertise. In IHC studies, the antibodies used for the evaluation can affect the obtained results [89,90]. Nonetheless, previous reports have successfully reported on the expression of SSTR1, SSTR2A, SSTR3, SSTR4, and SSTR5. In studies where all SSTRs have been assessed simultaneously, it has been demonstrated that SSTR4 is not expressed or is expressed in lower levels in NETs compared to other SSTRs [81,82,88]. The most expressed receptor subtypes are SSTR2A and SSTR5, following by a slightly lower abundance of SSTR3 and SSTR1 [81,82,88,91]. However, this information is still highly dependent upon the tumour type and methods used for the estimation of the expression level. Aside from the methodological differences described above, the subgrouping or classification of tumour types and preoperative SSA treatment can have a significant impact on the obtained results, which can hamper the generalisation of these findings in overarching conclusions (Table 1).

In many studies, all NET types are analysed together (Table 1), which is understandable given the rarity of these tumours. However, this can bias the results, since tumour development in specific tissue types causes intrinsic functional differences in tumour cells originating from specific cell types. Additionally, in many reports, the preoperative status of SSA is different; some studies include only SSA-treated patients, whereas others include both SSA-treated and naive cases without properly adjusting for the potential therapeutic impact (Table 1). It has been shown that SSA has the ability to downregulate SSTR expression, which means that an SSA pre-treatment can significantly affect SSTR expression [84,85].

Nonetheless, it has been widely proven that a higher SSTR expression is characteristic of well-differentiated NETs with tumour grades G1 or G2 [43,49,86,91,95,96]. Additionally, several reports have shown that NET patients with a higher tumour SSTR expression have improved survival [43,79,81,86,87,93,94]. Intriguingly, the better prognosis is also observed in those studies where subjects did not receive SSA therapy or only a small fraction of patients were treated with SSA [43,93,94]. This raises the question of how native SSTR expression without SSA therapy might contribute to better outcomes for NET patients. One plausible answer could be that an SSTR, in the absence of exogenous SSA, still receives endogenous somatostatin signals and this slows the tumour progression. It has been demonstrated that pancreatic NET metastases express lower levels of somatostatin, and the knockdown of somatostatin in pancreatic NET cell lines increases metabolic activity, viability, and growth [97].

In addition to providing prognostic insight on NET development, SSTR expression could also serve as a molecular determinant for predicting the SSA response for personalized therapy choices. This approach has been widely discussed for pituitary NETs, where SSTR expression levels are widely correlated with SSA treatment efficacy [98,99,100]. Other intrinsic tumour components such as genetic predisposition and somatic variation, expression pattern alterations and cellular patterns of dense or sparse granulation can be linked to the SSA response [99,101]. Recently, specific miRNA subtypes have also been shown to be dysregulated by SSA treatment or even downregulate SSTR, promoting tumour progression and indicating the presence of other crucial molecular markers in NETs [102].

The heterodimerization of SSTRs with dopamine receptors has also been widely demonstrated and has the potential to significantly affect NET pathophysiology and prognostics [103]. This has led to the development of chimeric somatostatin–dopamine agonists that could more effectively inhibit tumour progression [104]. Although the first generation of these chimeric compounds demonstrated promising results in preclinical studies, results from human studies were disappointing [103,105,106]. Currently, the second generation of chimeric agonists is under investigation and has shown positive effects both in cell lines and in healthy human trials [107,108,109,110]. Research in the field of chimeric somatostatin–dopamine agonists could bring additional improved therapeutic options for NET patients in the future.

Additionally, in recent years, epigenetic regulation in NETs has been implicated as a major tumourigenesis mechanism. For example, it has been demonstrated that the treatment of pancreatic NET cell lines treated with epigenetic modulators can result in the redifferentiation of human primary PanNETs. The upregulation of SSTR expression was observed in these studies, indicating that SSTR expression can be regulated by epigenetic tumour development mechanisms [111,112]. Specifically, valproic acid was used to upregulate SSTR2 expression and provide further benefit to SSTR-targeted therapies [113]. Other epigenetic agonists have been demonstrated to inhibit cell proliferation, reduce the progression and metastasis-forming capacity, induce apoptosis, and promote cytotoxic effects in various NET cell lines [114,115,116,117]. So far, despite promising evidence in preclinical settings, clinical trials have not confirmed the benefits in patient outcomes. A study conducted on eight patients showed that valproic acid has neutral to moderate effects, with an overall good tolerance to the treatment [118]. However, another study of 15 patients with metastatic NETs demonstrated that the histone deacetylase inhibitor depsipeptide was cardiotoxic [119]. In a separate study of 15 patients receiving Panobinostat, no significant benefits were demonstrated [120]. Taken together, these studies demonstrate that a further investigation of epigenetic agents is needed to determine the best strategy for improving NET control.

Additionally, in some studies, combination therapy using SSA with mTOR inhibitors showed some promising results. In future studies, it is crucial to also study signalling interactions of both pathways in NETs [121]. Overall, SSTR and somatostatin signalling is an important molecular factor that can affect the pathophysiology of NETs and should be considered as a target for SSA therapies for NET treatment.

## 5. Somatostatin Therapy, NET Treatment, and Internal Regulation

SST was initially viewed as a candidate for cancer treatment because of its ability to impede hormone release and cell growth after binding to its receptors. Unfortunately, the short half-life of native SST and the impact of rebound hypersecretion on discontinuation limited its use as a therapeutic agent. This prompted the development of clinically useful analogues with longer biological half-lives [122].

Somatostatin analogues have been produced by shortening the polypeptide chain and substituting two D-amino acids that are resistant to peptidase activity in circulating serum. The resulting molecule was a shorter cyclic peptide containing eight amino acids that retained the necessary amino acid moieties [3]. The first synthetic SSA to receive FDA approval was the octapeptide octreotide (SMS 201-995), marketed as Sandostatin^®^. It is available in two formulations: a conventional long-acting release (LAR) injection (Sandostatin LAR^®^) and modified long-acting release (LAR) injection (Sandostatin LAR^®^), which were approved in 1988 and 1998, respectively. Sandostatin LAR^®^ is an intramuscular injection formulation containing octreotide distributed within polymer microspheres. It is available in 30 mg doses. Octreotide binds to SSTR2 with a high affinity and inhibits cell growth via the activation of the tyrosine phosphatase pathway [7]. Camilleri’s group examined the effect of octreotide 50 mg subcutaneously three times daily on postprandial symptoms, the Gl transit, colonic motility, and circulating levels of selected peptides and amines in 12 individuals with multiple chemical sensitivity. Octreotide dramatically decreased the colonic transit time and proximal colonic emptying. In comparison to the placebo, it tended to lengthen the small-bowel transit time and decrease the postprandial colonic tone [123]. To test the safety and efficacy in an individual patient, SSA therapy should be initiated with 20 mg of Sandostatin LAR^®^ every 4 weeks. Patients who have a large tumour burden or those who develop insensitivity with time may require doses of up to 60 mg at 3–4 week intervals. Supplemental dosing with subcutaneous injections may be useful for breakthrough symptoms [3].

Lanreotide is a cyclic octapeptide that was created in the 1990s as a longer-acting SSA. Initially, one of its formulations (BIM23014) had a t½ of 90 min. Lanreotide sustained-release was introduced shortly thereafter, with a half-life of 4.5 days. It was constructed as biodegradable polymer microspheres that were administered intramuscularly in quantities of 30 or 60 mg every 7–14 days. Several years later, lanreotide Autogel^®^ (ATG) was introduced as a prefilled syringe for subcutaneous administration at a dose of 120 mg every 28 days [7]. Around 60% of patients showed a complete resolution of flushing episodes, and in more than 85%, the frequency and/or severity of flushing episodes was decreased to less than 50% of the baseline. More than 30% of patients showed a return to normal bowel movements with formed stools and more than 75% experienced more than a 50% reduction in stool frequency. Although both of these medicines have a low rate of objective tumour regression (2%), tumour stabilization is typical. Tumour progression is possible even when symptoms are managed, and, in this case, analogues were typically continued while additional therapy was added. Interferon has the ability to increase SSTR expression and reintroduce responsiveness in some patients who have become resistant to SSA. Abdominal discomfort, bloating, and, occasionally, steatorrhea are common side effects that resolve within a few days or weeks. Pancreatic enzyme supplements may help alleviate the steatorrhea associated with increased somatostatin analogue doses. In almost 50% of individuals, late side effects included the development of biliary sludge and gallstones. As a result, if the surgeon is in the abdomen performing a primary tumour resection or debulking disease in the liver, a preventive cholecystectomy should be performed. Steatorrhea that persists may result in a vitamin D insufficiency and calcium malabsorption [3].

Octreotide and lanreotide have similar binding characteristics and have a high affinity for SSTR2 and SSTR5, but have a low affinity for SSTR3, and no affinity for SSTR1 or SSTR4. Due to the fact that the absence of SSTR2 in tumours correlates with a lack of tumour response to octreotide, it has been postulated that octreotide exerts its effects primarily via SSTR2 [124]. In comparison to octreotide, pasireotide, a new SSA, has a 30-fold higher affinity for SSTR1, SSTR3, and SSTR5, and a 39-fold higher affinity for SSTR2. Additionally, pasireotide has a stronger affinity for SSTR1 (19-fold), SSTR3 (9-fold), and SSTR5 (106-fold), while having the same affinity for SST2R (2-fold). Pasireotide LAR (Signifor LAR^®^) was approved by the FDA in 2014 and is available in dosages of 20, 40, or 60 mg every 28 days through a intramuscular injection [7]. This new cyclic hexapeptide binds to SSTR1 30–40 times more strongly than Sandostatin (octreotide) or Somatuline (lanreotide). SSTR1 has been shown to stop the cell cycle and inhibit angiogenesis. Pasireotide was found to effectively treat diarrhoea and flushing in 25% of individuals with metastatic carcinoid tumours in a multicentre phase II clinical trial comprising 45 patients with symptoms of carcinoid syndrome who were unresponsive to Octreotide LAR. In 20–30% of patients, abdominal pain, nausea, weight loss, and exhaustion were mild to moderate. Pasireotide is being evaluated in clinical trials in carcinoid patients who have never been exposed to SSAs and in hormonally active pancreatic endocrine carcinomas [3].

## 6. Treatment with SSA Compared to Other Therapies, and the Continued Relevance of SSAs in the Treatment of NETs

SST is a pluripotent hormone that is widely utilized in the treatment of a variety of illnesses, including those not listed on the drug datasheet (off-label). SSAs are mostly utilized to diagnose and treat well-differentiated GEP-NETs as a first-line therapy, as well as to treat acromegaly. SSAs are also used in the disciplines of endocrinology, cancer, digestive, general surgery, and ophthalmology due to their considerable antisecretory, direct and indirect antiproliferative, and immunomodulatory activity [7]. Although there is no information in guidelines regarding the use of SSAs as adjuvant therapy, postoperative treatment with SSAs can prolong disease-free survival and lower the risk of tumour recurrence [125]. Adjuvant therapy following surgery may be considered in the context of aggressive or G3 tumours [126]. The goal of systemic therapy is to control the tumour-associated clinical symptoms and the tumour growth.

In many individuals with NETs, neuropeptide hypersecretion accounts for the majority of clinical issues. The carcinoid syndrome (CS) was first documented in a case report and has since been linked to the tumour production of serotonin (5-hydroxytryptamine) and histamine [8]. A carcinoid crisis can occur spontaneously or be triggered by physical and/or psychological stress, alcohol, meals high in tyramine, or anaesthesia. The most prevalent symptom (85%) is the abrupt flushing of the face, neck, and upper thorax, which may be accompanied by feeling heat, pruritus, tachycardia, or arterial hypotension. As the condition advances, flushing episodes become more intense and frequent, and the skin can permanently acquire a pinkish hue due to telangiectasias. Diarrhoea and stomach pain are present in 80% of cases and may be accompanied by distinct flushing symptoms. Bronchospasm and dyspnoea occur less frequently (15%), but often coincide with flushing episodes. Carcinoid heart disease is characterized by fibrotic lesions that are most frequently found in the right heart. Additional symptoms include pellagra, weakness, and muscle atrophy [7]. While serotonin is responsible for diarrhoea and fibrosis, co-secreted peptides such as histamine, bradykinins, and tachykinins are responsible for flushing and respiratory symptoms. Although curative surgery is possible in neuroendocrine tumours, CS is frequently coupled with liver metastases, which render treatment choices non-curative in the majority of cases [8].

Palliative therapeutic options include SSAs, interferon alpha (IFNα), chemotherapy, locoregional therapies, molecularly targeted medicines, and peptide receptor radionuclide therapy (PRRT), according to current guidelines. Though these medicines frequently fail to halt tumour growth, they are often able to improve a patient’s health-related quality of life by alleviating CS symptoms. Notably, a recent meta-analysis found that octreotide significantly reduced diarrhoea and flushing in 65% and 72% of patients, respectively. Notably, lanreotide had striking similarities in its effects. In patients who were unresponsive to normal SSA first-line treatment, dosage escalation, or shortening the injection interval to 21 days led to a 72% reduction in diarrhoea and an 84% reduction in flushing. As previously stated, patients with CS are at risk for developing carcinoid cardiac heart disease (CHD). CHD is characterized by endocardial and valve leaflet fibrosis in the right heart. Because CHD is more prevalent in people with elevated serotonin and 5-HIAA levels, SSA medication was suggested as a treatment option. In support of this concept, a recent trial indicated that SSA therapy reduces the risk of CHD in patients with CS [8]. In addition to reducing hormone production by NETs, SSAs have been reported to reduce upper abdominal pain, improve the quality of life and performance status, promote the healing of pancreatic fistulae, and improve orthostatic hypotension [122]. In GNETs, long-acting SSAs can be employed to block both the gastrin release and endocrine cell proliferation directly. SSA treatment has been shown to decrease and even normalize gastrin and CgA levels, as well as promote GNET-1 regression [127].

IFNα is a type of cytokine that has antiviral, antiproliferative, and antitumour properties. Since 1982, it has been used to treat metastatic NETs alone or in combination with chemotherapy and SSAs. IFNα is administered subcutaneously in doses ranging from 3 to 5 million units (mU) three times per week, or as weekly injections of 75–150 g long-acting pegylated (PEG)-IFNα. Initial flu-like symptoms, chronic exhaustion, depression, anaemia, and neutropenia are all possible side effects. Additionally, 15–20% of patients exhibit autoimmune responses, the most prevalent of which is thyroid dysfunction [128].

Apart from IFNα, pasireotide has been tested recently as a second-line therapy in CS patients who are resistant to SSA. In 27% of these patients, pasireotide resulted in symptom control [8]. Although a preliminary study supported the use of pasireotide in the treatment of patients with metastatic carcinoid syndrome who were resistant to conventional SSAs, a phase III study demonstrated that pasireotide LAR and high-dose octreotide LAR had a comparable efficacy for symptom control in patients with functional NETs and symptoms that were not adequately controlled with the recommended doses of available SSAs. Furthermore, it is unknown whether pasireotide is more antiproliferative than octreotide/lanreotide [129]. Pasireotide’s toxicity profile is also less favourable, as it has been associated with a higher incidence of hyperglycaemia, in addition to the gastrointestinal symptoms (primarily abdominal pain, nausea, and diarrhoea) observed with first-generation SSAs. As a result, pasireotide is not currently considered a standard of care for patients with NETs. Its hyperglycaemic properties may be significant in certain cases of insulinoma and in NETs with distinct SSTR profiles [8]. Additionally, the efficacy of combination therapy with pasireotide and everolimus in NETs is debatable. Although studies examining the combination of pasireotide and everolimus demonstrated a higher response rate in terms of tumour stabilization and regression, this improved response rate did not result in a statistically significant improvement in progression-free survival when compared to either drug alone [129]. At the moment, only octreotide and lanreotide are available as SSAs with clinical evidence of antitumour efficacy in a phase III trial setting [130].

Everolimus research in the treatment of NETs is particularly active at the moment, with a number of studies currently underway or awaiting final results. Furthermore, everolimus is being studied in combination with other targeted therapies such as sorafenib, bevacizumab, or temozolomide. An intriguing strategy that our group is actively pursuing is the combination of everolimus and metformin, owing to the potential antiproliferative effect of everolimus and its ability to control hyperglycaemia. Additionally, the combination of everolimus and PRRT is being investigated at the moment [131].

Telotristate etiprate was recently developed as a novel inhibitor of tryptophan hydroxylase, the rate-limiting enzyme in the biosynthesis of serotonin. This medication is capable of suppressing serotonin production in patients with NETs, as evidenced by a decrease in u5HIAA, while concurrently improving diarrhoea to some degree [132]. Numerous adverse effects have been reported, including nausea, headache, elevated liver enzymes, depression, peripheral oedema, flatulence, a decreased appetite, and fever. All of these side effects are reversible and manageable in the majority of patients following treatment [8].

SSAs can also inhibit the growth of NETs. Clinical trials have shown that SSAs can halt tumour progression, but patients rarely have objective tumour regression. SSAs work both directly and indirectly to control tumour growth. The direct antimitotic effect is mediated by somatostatin receptors on tumour cells. Indirect effects of SSAs, such as the inhibition of growth factor secretion, inhibition of angiogenesis, and immunomodulatory effects on peripheral target organs, also contribute to tumour control. By suppressing the synthesis and secretion of growth factors, including the insulin-like growth factor (IGF-1), an important modulator of many neoplasms, octreotide is able to exert antiproliferative effects and reduce tumour growth [122]. Moreover, SSA treatment decreased microvessel development by 50% and it was shown that SSA works on VEGF through blocking transcription factor recruitment, which may be related transcriptional activity of SF3B1 [133]. Compared with native somatostatin, octreotide and pasireotide are able to inhibit neovascularization to a greater extent, possibly through interactions with peritumoural vascular SSTR2 receptors. SSAs may also exert antiangiogenic effects through the inhibition of growth factors (e.g., PDGF, IGF-1, and the epidermal growth factor), which are known to stimulate important processes in angiogenesis, such as endothelial and smooth muscle cell proliferation. Finally, because SSTRs are expressed on various cells of the immune system (e.g., lymphocytes and monocytes), octreotide may regulate inflammatory and immune mechanisms to possibly enhance its antiproliferative activity [122].

SSTR2 and SSTR5 expression at high levels on the surface of the majority of NETs enables not only sensitive functional imaging, but also tumour-targeted therapy with commonly used “cold” and radioisotope-labelled “hot” somatostatin analogues. Apart from cold somatostatin analogues as a first-line antiproliferative therapy, PRRT has emerged as a highly effective treatment option for metastatic, well-differentiated GEP-NETs of low and intermediate grades (G1 and G2) in recent decades [8]. In 1992, the first PRRT was carried out in Rotterdam with 111In-DTPA-octreotide (OctreoScan^®^), a well-known functional imaging tracer with a short path length and gamma radiation release. The initial objectives, which included a reasonable response and an extraordinary suppression of carcinoid symptoms, were met [134].

The next generation of beta-emitting agents developed was the 90Y radionuclide conjugated to DOTA peptides (DOTATATE and DOTATOC). Frequently, the objective response and prolongation of progression-free survival (PFS) could be demonstrated, though a careful monitoring of the kidneys was recommended. Due to radionuclide deep tissue penetration (12 mm) and a physical half-life of 64 h, significant damage to the kidney glomeruli and bone marrow was a frequent side effect. A total of 12.8% of the 1109 patients treated with 90Y-DOTATOC developed transient hematologic toxicities of grade three or four, and more than 9% developed grade four or five permanent renal toxicity. As a result, an amino acid co-infusion with lysine/arginine was introduced as a renal protective regimen. Due to the numerous advantages of 177Lu (path length 2 mm; t½ 6.7 days), in recent years, 90Y has been largely phased out. Minor adverse effects, such as abdominal pain, were reduced to less than 1% depending on the location of metastases, and a remarkable reduction in adverse sequelae (e.g., nephrotoxicity) was achieved [8]. Additionally, 177Lu is an α-emitter, which enables visualization, staging, and dosimetry via single-photon emission computed tomography (SPECT/CT) [135]. Numerous retrospective studies examining the feasibility, outcome, and safety of PRRT also demonstrated a PFS comparable to, and in some cases superior to, that of other treatment modalities. Due to the retrospective nature of these studies, the results required confirmation in prospective trials. According to PanNET guidelines, PRRT should even be used as a third-line therapy in patients with advanced locoregional disease following the failure of SSAs, everolimus, and/or cytotoxic chemotherapy [8]. Two cases were presented for third-line therapy, in which patients with metastatic PanNET (G2 Ki-67 10%) received chemotherapy or PRRT according to the standard of care following SSA failure. After six cycles of STZ/5-FU (over nine months), the patient progressed four months later without treatment. The majority of participants preferred PRRT (38.6%, n = 32) over STZ/5-FU reinduction (26.5%, n = 22) [136]. In patients with GEP-NET G1 and G2, 177Lu-PRRT was compared to the mTOR inhibitor everolimus. Despite the success of 177Lu-DOTATATE PRRT, not all patients showed benefits, and patients typically relapsed 2–3 years after initiating treatment. As a result, various experimental approaches and strategies are being investigated in order to maximize PRRT’s effectiveness while minimizing potential side effects [8]. The NETTER-1 phase III study demonstrated that, in addition to improving progression-free survival, 177Lu-Dotatate significantly improves patient quality of life significantly when compared to high-dose octreotide [137]. In the study where 229 patients randomly received Lu-DOTATATE every eight weeks for four cycles plus 30 mg octreotide every four weeks or high-dose octreotide (60 mg) every four weeks alone showed a significant reduction in tumour progression or death in the Lu-DOTATATE treatment group. According to recent investigations, the time to quality of life decline was substantially longer in the 177Lu-DOTATATE therapy group. After the NETTER-1 phase III trial, the 177Lu-DOTATATE treatment was approved for the somatostatin receptor-positive GEP-NETs [138]. To begin, treatment may be continued after four cycles of PRRT, depending on kidney and bone marrow tolerance, for example, with a reduced radioactivity as part of the salvage therapy (Re-PRRT). Retreatment with 177Lu-DOTATATE-based PRRT showed an optimistic survival benefit with acceptable safety in patients with NETs [139]. The administration of PRRT intra-arterially into the hepatic artery rather than intravenously may increase the tumour-absorbed dose in liver metastases. However, intra-arterial administration is not mentioned in guidelines and is considered experimental [140]. Patients with hepatic dominant metastases, in particular, would benefit from this approach due to the increased uptake of the radiopharmaceutical (the so-called “first-pass” effect). In a neoadjuvant setting, the PRRT is becoming increasingly important. PRRT was associated with a significant reduction in tumour size and the tumour was rendered operable in patients with inoperable PanNETs and distant (metastatic) disease. In such instances, a complete response was possible [8]. PRRT was likely to be the best treatment option in patients with advanced well-differentiated NETs in terms of the benefit–risk ratio. Numerous nonrandomized studies of PRRT have consistently demonstrated high response rates and prolonged PFS in patients with GEP-NETs. In a more recent randomized phase III trial, PRRT plus the best supportive care provided a longer PFS than high-dose octreotide, and did so with limited serious adverse events (SAEs) in patients with advanced midgut NETs. However, PRRT has not been directly compared to any other established regimen. PRRT outperformed SSA, everolimus, sunitinib, everolimus + SSA, and everolimus + bevacizumab in terms of PFS. PRRT had the highest probability (96%) of being the most effective treatment in terms of improving PFS and posing a low risk of SAEs based on cluster ranking [129].

Only SSTR agonists have been labelled with beta-emitters thus far. SSTR antagonists are expected to have a higher affinity for somatostatin-positive tumour cells, resulting in an increase in the radiation dose delivered to the tumour. Another intriguing approach is to use alpha emitters such as Bismuth-213 (tissue penetration 45 μm, t½ 45 min) or Actinium-225 (tissue penetration 45 μm, t½ 10 d) as a targeted alpha particle therapy (TAT). TAT treatment has gained popularity in recent years, particularly in the treatment of the castration-resistant prostate cancer-expressing 177Luprostate-specific membrane antigen. The advantage, it has been hypothesized, is a shallow tissue penetration depth combined with a high dose of ionizing radiation. The next logical step is to apply these promising results to TAT in NETs. However, evidence for TAT is still sparse at the moment [8]. Zhang et al. recently reported another promising development involving the novel SSTR agonist DOTA-EB-TATE, which was also labelled with 177Lu. In comparison to octreotide and octreotate, this agonist demonstrated a significantly greater uptake and retention in NETs. The authors conclude that it may be used in the future in PRRT for NETs due to its lower dose and less frequent administration [141].

## 7. Conclusions

In this review, we aimed to provide a comprehensive overview of SST in NET development and treatment to present overarching information for researchers in this and other fields of research. In conclusion, SST signalling has a crucial role in NET pathogenesis. SSTRs are important drug targets for NET treatment regimens. SSTR expression has a prognostic value not only for treatment outcomes, but also for evaluating patient-specific survival prognosis. Different SSA therapy modalities will remain relevant treatment strategies, though in the future, it seems likely that novel therapeutic combinations will be utilized.

## Figures and Tables

**Table 1:** Studies investigating SSTR expression in NETs and the potential impact of SSTR expression on disease prognostics.

Publication	Type and Number of the Studied Tumours	Preoperative Therapy with SSA	Receptors	Method	Correlation with Disease Outcome, Prognosis, or Other Relevant Findings
Brunner et al., 2016 [79]	PanNETs (n = 150, 53.8%), carcinoids (n = 84, 30.1%), and rare NETs (n = 45, 16.1%)	DOTATOC 41 (24.1%) DOTATOC 84 (49.4%) DOTATOC 45 (26.5%)	SSTR2	IHC and imaging for all DOTATOC patients	-Patients with higher SSTR2 immunohistochemistry had a longer survival from diagnosis -Patients with G1 tumours survived longer thanks to other tumour grades -SSTR2 expression did not predict a benefit of DOTATOC over alternative treatment
Casar-Borota et al., 2013 [80]	Somatotroph PitNETs (n = 65)	Octreotide (n = 28); no preoperative treatment (n = 37)	SSTR1, SSTR2A, SSTR3, SSTR5	IHC	-SSTR2a expression correlated with response to octreotide -SSTR2a expression was reduced in group of patients pre-treated with octreotide
Corleto et al., 2009 [81]	Well-differentiated PanNETs (n = 16, 49%) and well-differentiated GI-NET (n = 17, 51%)	All with SSA (octreotide LAR or lanreotide)	SSTR1, SSTR2, SSTR3, SSTR4, SSTR5	RT-PCR (SSTR1, SSTR2, SSTR3, SSTR4, and SSTR5), IHC (SSTR2), and somatostatin receptor scintigraphy	-Significantly higher survival rate in tumours expressing SSTR2 and SSTR5 -Five-year survival significantly lower in patients whose tumours did not express SSTR2, SSTR5 (43%) to those who were positive for the expression (91%)
Diakatou et al., 2011 [82]	GEP-NETs (n = 76: gastric, small intestine, appendix, large intestine, pancreas, and liver metastasis)	No information	SSTR1, SSTR2A, SSTR2B, SSTR3, SSTR4, SSTR5	IHC	-No information related to prognosis -SSTR are often co-expressed with D2R
Diakatou et al., 2015 [83]	GI-NENs (n = 44, primary and metastasis) and Lu-NENs (n = 16, primary and metastasis)	No information	SSTR2, SSTR3, SSTR5	SRS, IHC	-No information related to prognosis -IHC positivity correlated with SRS -D2R were always co-expressed with SSTR2, but not only in specific fraction of tumours expressing SSTR2R
Fougner et al., 2008 [84]	Somatotroph PitNETs (n = 71)	Twenty-three preoperative octreotide	SSTR2A	IHC, Western blot	-Patients with preoperative octreotide treatment had lower SSTR2a expression in IHC and Western blot -Acute octreotide response was significantly better comparing treatment-naïve and pre-treated patients having higher SSTR2A expression in IHC
Franck et al., 2017 [85]	Somatotroph PitNETs (n = 39)	Drug naive (n = 23); pre-treated with LA SSA (n = 9); LA-SSA/PEGV (n = 7)	SSTR2, SSTR5	IHC	-Treatment-naïve patients had significantly higher SSTR2 expression compared to pre-treated patients -No differences were observed for SSTR5 expression
Kaemmerer et al., 2015 [86]	GI-NENs (n = 121) G1 (n = 31), G2 (n = 47), and G3 (n = 43)	No information	SSTR1, SSTR2A, SSTR3, SSTR 5	IHC	-Expression of SSTR2 was higher in G1 and G2 tumours -SSTR2A expression demonstrated trend towards better overall survival -SSTR1 expression was significantly different in G2 compared to G3b (highly proliferative Ki-67 > 50%) -SSTR3 expression was significantly different in G2 compared to G3a (low proliferative Ki-67 21–49%)
Mehta et al., 2015 [87]	PanNETs (n = 99)	No information	SSTR2A, SSTR5	IHC	-SSTR2A expression correlated with improved overall survival -SSTR5 was not associated with survival
Nasir et al., 2006 [92]	Liver metastasis from patients with small intestinal and Pan-NETs (n = 14)	All with octreotide therapy	SSTR1, SSTR2, SSTR3, SSTR4, SSTR5	IHC	-No information related to prognosis -In total, 61% of studied metastasis was positive for SSTR1, 83% for SSTR2, 72% for SSTR3, 56% for SSTR4, and 83% for SSTR5
Nielsen et al., 2020 [93]	GEP-NENs (n = 163)	Two patients received peptide receptor radionuclide therapy	SSTR2A	IHC	-Tendency for increased survival in patients with tumour with positive SSTR2a expression
Papotti et al., 2002 [88]	Total GEP NET (n = 81: GI (n = 28) and PanNETs (n = 53))	No information	SSTR1, SSTR2, SSTR3, SSTR4, SSTR5	RT-PCR (SSTR1, SSTR2, SSTR3, SSTR4, and SSTR5) and immunohistochemistry (SSTR2, SSTR3, and SSTR5)	-No information related to prognosis -Results from RT-PCR and IHC were comparable -Tendency that lower level SSTR expression in poorly differentiated tumours
Okuwaki et al., 2013 [94]	PanNETs (n = 79)	Fifty-nine patients’ surgery as first line treatment, and the rest that accepted treatment any of the following regiments (SSA, chemotherapy, and local targeting of liver metastasis)	SSTR2A	IHC	-Poor prognosis was predicted for patients with no SSTR2A expression -Higher SSTR2A expression predicted higher survival rate
Popa et al., 2021 [49]	GI-NENs (n = 67)	No information	SSTR2, SSTR5	IHC	-Tumours with grade G1 and G2 had higher SSTR2 expression compared to G3 -Decreased SSTR2 expression was associated with increased malignancy and tumour stage
Righi et al., 2010 [95]	Lu-NETs (n = 218)	Eight patients octreotide or DOTATOC	SSTR2A, SSTR3,	IHC	-Higher grade tumour had lower levels of SSTR expression; -SSTR2A was overexpressed in metastatic typical carcinoid compared to atypical carcinoids
Srirajaskanthan et al., 2009 [96]	NETs (n = 56: foregut (n = 20), midgut (n = 25), hindgut (n = 3), ovarian (n = 1), and unknown primary origin (n = 7))	No information	SSTR2, SSTR5	IHC	-Lower grade tumours had significantly higher SSTR2 and SSTR5 expression -OctreoScan data were comparable with IHC
Wang et al., 2017 [43]	GEP-NETs (n = 143) and GEP-NET patients with octreotide LAR (n = 54)	GEP-NET octreotide LAR (n = 54)	SSTR2, SSTR5	IHC	-G1 and G2 tumours had higher SSTR2 expression -Well-differentiated pancreatic NETs had higher SSTR5 expression -SSTR2 and SSTR5 expression in GEP-NETs were correlated with improved survival
Zamora et al., 2010 [91]	GEP-NETs (n = 100: GI (n = 67), pancreatic (n = 25), and liver metastasis of unknown origin (n = 8)	No information	SSTR1, SSTR2, SSTR3, SSTR4, SSTR5	IHC	-Well-differentiated NETs had higher SSTR expression; -SSTR expression was less frequent in pancreatic NETs compared to gastrointestinal NETs -SSTR2A was expressed on call membrane, while other subtypes (SSTR 1, 3, 4, 5) stained in cytoplasm

NET, neuroendocrine tumour; NEN, neuroendocrine neoplasia; PanNET, pancreatic NET; PitNET, pituitary NET; GI-NET, gastrointestinal NET; GEP-NET, gastroenteropancreatic NET; Lu-NET, lung NET; SSTR, somatostatin receptor; IHC, immunohistochemistry; SRS, somatostatin receptor scintigraphy; RT-PCR, reverse transcription polymerase chain reaction.

## Data Availability

Not applicable.
